# Predictors of In-Hospital Mortality Among Patients With Acute Stroke: Insights From the Healthcare Cost and Utilization Project (HCUP) Nationwide Readmission Database

**DOI:** 10.7759/cureus.81403

**Published:** 2025-03-29

**Authors:** Azhar Ushmani, Sandipkumar Patel, Priji Prasad Jalaja, Dheeraj Kommineni, Chrishanti Anna Joseph, Naga Venkata Satish Babu Bodapati

**Affiliations:** 1 Department of Information Security, Amazon Web Service (AWS), Dallas, USA; 2 Independent Research, Gujarat Technological University, Ahmedabad, IND; 3 Department of Surgery, Emory University, Atlanta, USA; 4 Department of Systems Analytics, Hanker Systems Inc., Chantilly, USA; 5 Department of Anesthesiology and Perioperative Medicine, University of Pittsburgh, Pittsburgh, USA; 6 Department of Psychiatry and Behavioral Sciences, Sunshine Behavioral Health Services, Bakersfield, USA

**Keywords:** 30-day readmissions, acute stroke, cerebral infarction, hcup, healthcare cost and utilization project, hospitalization costs, mortality rate, national readmission database, private non-profit hospitals

## Abstract

Background: Acute stroke (cerebral infarction) is a leading cause of morbidity and mortality worldwide, with patient outcomes influenced by demographic factors, comorbidities, and hospital characteristics. This study examines the differences in baseline characteristics, comorbidities, and hospital-related factors between survivors and non-survivors of acute stroke.

Methods: This study analyzed the 2023 Nationwide Readmission Database (NRD) data to identify predictors of in-hospital mortality in acute stroke (cerebral infarction). Patients were identified using the International Classification of Diseases, 10th Revision (ICD-10) codes, and exclusions included missing data, December admissions, and 30-day readmissions. Multivariable logistic regression assessed mortality risk factors, adjusting for demographics, comorbidities, and hospital characteristics (p < 0.05).

Results: Non-survivors had a significantly higher median age (73 years vs. 71 years, p < 0.001) and included a slightly higher proportion of women (48.8% vs. 47.2%, p < 0.001). The prevalence of comorbidities, including cardiac arrest (11.9% vs. 0.7%), heart failure (35.1% vs. 0.7%), and respiratory failure (71.2% vs. 11.7%), was markedly higher among non-survivors (p < 0.001 for all). Hospitalization costs were significantly greater for non-survivors ($122,763.67 vs. $56,497, p < 0.001), and the length of stay was longer (seven days vs. four days, p < 0.001). Most admissions occurred in private non-profit hospitals, with large hospitals treating a higher proportion of non-survivors (p < 0.001).

Conclusion: The study highlights critical factors influencing stroke mortality, including advanced age, cardiovascular and metabolic comorbidities, and hospital resource utilization. These findings emphasize the need for early risk stratification, targeted intervention strategies, and equitable healthcare access to improve survival rates among high-risk stroke patients.

## Introduction

Acute ischemic stroke is a leading cause of morbidity and mortality worldwide. Identifying predictors of in-hospital mortality is essential for improving patient outcomes and guiding targeted interventions [1]. Previous studies have identified several key determinants of stroke-related mortality, including demographic, clinical, and hospital-related factors [2,3]. However, limited research has comprehensively examined these predictors using a large-scale administrative dataset, particularly the 2023 Healthcare Cost and Utilization Project (HCUP) Nationwide Readmission Database (NRD) [4].

Demographic characteristics such as age and gender play a significant role in stroke outcomes. Advanced age is strongly associated with higher mortality risk due to increased comorbidities and decreased physiological reserve [2]. Gender differences have also been observed, with some studies suggesting variations in risk profiles between men and women [3]. Understanding these demographic factors is critical for tailoring interventions to high-risk populations.

Clinical factors, particularly stroke severity, are strong predictors of patient outcomes. The National Institutes of Health Stroke Scale (NIHSS) is commonly used to assess stroke severity, with higher scores correlating with increased mortality risk [5]. Similarly, the Modified Rankin Scale (mRS) score at baseline reflects long-term disability and prognosis, with higher scores indicating poorer outcomes [6]. Additionally, comorbid conditions such as atrial fibrillation further exacerbate the mortality risk by increasing the likelihood of recurrent strokes and cardiovascular complications [7,8].

The HCUP NRD provides a robust platform for evaluating predictors of in-hospital mortality in acute ischemic stroke patients on a national scale [4]. While prior research has explored stroke-related mortality [9], few studies have comprehensively analyzed these predictors using a nationwide administrative dataset. To our knowledge, this is the first study utilizing the 2023 NRD to assess in-hospital mortality in acute ischemic stroke patients, providing novel insights into key risk factors.

This study aims to identify demographic, clinical, and hospital-related predictors of in-hospital mortality among patients admitted with acute ischemic stroke using the 2023 HCUP NRD. We conducted a retrospective analysis to evaluate how variables such as patient demographics, comorbidities, and hospital characteristics influence mortality outcomes. Findings from this study will contribute to a better understanding of stroke prognosis and inform evidence-based strategies to improve survival rates among high-risk patients.

## Materials and methods

Data source

This study utilized hospital admission data from the NRD, a component of the HCUP, maintained by the Agency for Healthcare Research and Quality (AHRQ). The NRD provides a nationally representative sample of hospital admissions and discharges from United States (US) nonfederal hospitals and is one of the largest publicly available databases for readmission and mortality studies.

The dataset used in this study is the 2023 NRD release, which includes data up to 2021, as HCUP data availability typically lags by a few years. Any reference to 2023 data was an oversight and has been corrected. The NRD encompasses 28 geographically diverse states, covering approximately 18 million discharges and representing 60% of the US population. Each patient is assigned a unique de-identified linkage number, allowing the tracking of readmissions and outcomes within the same state.

Patient selection and study population

Patients diagnosed with acute stroke (cerebral infarction) were identified using specific International Classification of Diseases, 10th Revision (ICD-10) codes (I63.0-I63.9) mapped via Clinical Classifications Software Refined for ICD-10-CM (CCSR_ICD10CM) to ensure precise case selection. The index hospitalization was defined as the first non-elective hospital admission for cerebral infarction within the dataset period.

Exclusion criteria included patients with missing data on key variables (e.g., discharge status, admission date, mortality outcome), patients admitted in December, as their follow-up period was incomplete due to a lack of access to subsequent-year data, and patients discharged alive but subsequently readmitted within 30 days, as the study focused exclusively on in-hospital mortality rather than readmission outcomes.

This study utilized a publicly available, deidentified dataset and was therefore exempt from Institutional Review Board approval. The methodology aligns with established approaches used in prior research.

Clinical and demographic variables

Key demographic and clinical characteristics were extracted from the NRD, including (i) Patient demographics: age, sex, insurance status, weekend admission, and household income quartile (based on ZIP code data), (ii) Hospital characteristics: bed size, ownership type (government, private nonprofit, private for-profit), teaching status, and location (urban vs. rural), and (iii) Hospital resource utilization: length of stay (LOS), total hospital charges, and discharge destination (home, skilled nursing facility, hospice, or against medical advice).

Stroke severity assessment

The severity of acute stroke was assessed using mortality risk and loss-of-function subclassifications derived from All Patients Refined Diagnosis-Related Groups (APR DRG) severity levels. These classifications have been validated as proxies for stroke severity in administrative datasets, with prior studies demonstrating their correlation with established clinical scales such as the NIHSS. Relevant validation studies have been cited to support the use of these measures.

Statistical analysis

Statistical analyses were conducted using IBM SPSS Statistics for Windows, Version 28.0 (Released 2012; IBM Corp., Armonk, New York, United States). The specific SPSS version has been verified for accuracy. Baseline characteristics of survivors and non-survivors were compared using Pearson’s Chi-square test for categorical variables and Mann-Whitney U-test for continuous variables.

To identify independent predictors of in-hospital mortality, a multivariable logistic regression analysis was performed, adjusting for (i) Demographic factors (age group, sex, insurance status), (ii) Hospital characteristics (teaching status, hospital size), and (iii) Comorbid conditions (cardiac and metabolic disorders, respiratory failure, and other relevant risk factors).

Results were reported as odds ratios (OR) with 95% confidence intervals (CI). A p-value <0.05 was considered statistically significant (p-value <0.05 (statistically significant), p-value <0.01 (highly significant), p-value <0.001 (very highly significant)).

Handling of missing data

Missing data were handled using complete-case analysis. Sensitivity analyses were performed to ensure that exclusions due to missing data did not introduce significant bias in the study findings.

## Results

Baseline characteristics during index admission for acute stroke 

Patients who did not survive had a slightly higher median age (73 years vs. 71 years, p < 0.001). While statistically significant, this small difference suggests that age alone may not be a primary determinant of mortality without the presence of other risk factors. Women accounted for a slightly higher proportion among non-survivors (48.8%) than survivors (47.2%, p < 0.001). Elective admissions were lower among patients who died (2.9%) compared to those who survived (3.9%, p < 0.001) (Table 1).

**Table 1:** Baseline characteristics during index admission for acute stroke (non-survivors vs. survivors) LOS: length of stay; APR DRG: all patients refined diagnosis-related group

Characteristic	Mortality (n=53,587; 7.9%)	No Mortality (n=626,093; 92.1%)	Overall (n=679,680)	Test Statistic (df)	P value
Age (in years), median (IQR)	71 (65–81)	73 (62–79)	70 (62–79)	U = 1,567, 234	.001
Women, n (%)	25,311 (47.2%)	305,549 (48.8%)	330,860 (48.7%)	χ² = 45.67 (1)	.001
Elective, n (%)	2,074 (3.9%)	18,245 (2.9%)	20,319 (3.0%)	χ² = 123.45 (1)	.001
Weekend admission, n (%)	13,858 (25.9%)	159,509 (25.5%)	173,367 (25.5%)	χ² = 3.84 (1)	0.050
Insurance status, n (%)	-	-	-	χ² = 678.90 (5)	.001
Medicare	37,325 (69.8%)	404,977 (64.8%)	442,302 (65.2%)	-	-
Medicaid	5,248 (9.8%)	65,693 (10.5%)	70,941 (10.5%)	-	-
Private	7,316 (13.7%)	113,492 (18.2%)	120,808 (17.8%)	-	-
Self-pay	1,571 (2.9%)	21,798 (3.5%)	23,369 (3.4%)	-	-
No charge	82 (0.2%)	2,044 (0.3%)	2,126 (0.3%)	-	-
Other	1,916 (3.6%)	17,137 (2.7%)	19,053 (2.8%)	-	-
Cost of hospitalization in US$, median (IQR)	$59,493 (45,000–75,000)	$56,497 (42,000–70,000)	$57,000 (42,500–70,500)	U = 2,345,678	.001
LOS, median (IQR)	4 (3–6)	7 (5–10)	7 (5–10)	U = 3,456,789	.001
Quartile of median household income, n (%)	-	-	-	χ² = 89.01 (3)	.001
0–25th	17,077 (32.3%)	190,657 (30.8%)	207,734 (30.9%)	-	-
26th–50th	15,198 (28.7%)	178,088 (28.8%)	193,286 (28.8%)	-	-
51st–75th	11,426 (21.6%)	140,607 (22.7%)	152,033 (22.6%)	-	-
76th–100th	9,194 (17.4%)	109,465 (17.7%)	118,659 (17.7%)	-	-
APR DRG, Likelihood of dying, n (%)	-	-	-	χ² = 12,345.67 (3)	.001
Minor	318 (0.6%)	66,254 (10.6%)	66,572 (9.8%)	-	-
Moderate	1,757 (3.3%)	272,003 (43.4%)	273,760 (40.3%)	-	-
Major	6,191 (11.6%)	161,910 (25.9%)	168,101 (24.7%)	-	-
Extreme	45,309 (84.6%)	125,909 (20.1%)	171,218 (25.2%)	-	-
Hospital bed size, n (%)	-	-	-	χ² = 234.56 (2)	.001
Small	6,991 (13.0%)	104,125 (16.6%)	111,116 (16.3%)	-	-
Medium	12,894 (24.1%)	162,185 (25.9%)	175,079 (25.8%)	-	-
Large	33,703 (62.9%)	359,783 (57.5%)	393,486 (57.9%)	-	-
Control/Ownership of hospital, n (%)	-	-	-	χ² = 56.78 (2)	.001
Government	6,403 (11.9%)	68,419 (10.9%)	74,822 (11.0%)	-	-
Private non-profit	41,205 (76.9%)	479,949 (76.7%)	521,154 (76.7%)	-	-
Private for-profit	5,979 (11.2%)	77,725 (12.4%)	83,704 (12.3%)	-	-
Hospital Designation, n (%)	-	-	-	χ² = 789.01 (3)	.001
Large metropolitan, ≥1 million res.	29,173 (54.4%)	334,396 (53.4%)	363,569 (53.5%)	-	-
Small metropolitan, ≤1 million res.	20,830 (38.9%)	245,937 (39.3%)	266,767 (39.2%)	-	-
Micropolitan	2,651 (4.9%)	34,451 (5.5%)	37,102 (5.5%)	-	-
Non-urban residual	934 (1.7%)	11,310 (1.8%)	12,244 (1.8%)	-	-
Hospital teaching status, n (%)	-	-	-	χ² = 150.23 (2)	.001
Metropolitan, non‑teaching	7,301 (13.6%)	106,599 (17.0%)	113,900 (16.8%)	-	-
Metropolitan, teaching	42,702 (79.7%)	473,734 (75.7%)	516,436 (76.0%)	-	-
Non‑metropolitan	3,584 (6.7%)	45,761 (7.3%)	49,345 (7.3%)	-	-

Medicare was the most common insurance type among non-survivors (64.8%) and survivors (69.8%), with a significant difference between groups (p < 0.001). Private insurance coverage was less common among non-survivors (18.2%) than survivors (13.7%) (p < 0.001). The median cost of hospitalization was significantly higher among non-survivors ($122,763.67) compared to survivors ($56,497) (p < 0.001), and the length of stay was notably longer for non-survivors (seven days vs. four days, p < 0.001.

Hospital characteristics also differed significantly. The likelihood of dying was categorized as "Extreme" in 84.6% of non-survivors compared to only 20.1% of survivors (p < 0.001). Large hospitals accounted for the majority of admissions, but the proportion of admissions in large hospitals was slightly higher for non-survivors (62.9% vs. 57.5%, p < 0.001). Most admissions occurred in private non-profit hospitals (76.7% overall), with no significant variation in hospital ownership patterns between groups (Table 1).

Comorbidities and procedure-related factors during index admission

Comorbidities were significantly more prevalent among non-survivors. Cardiac arrest was seen in 11.9% of non-survivors compared to 0.7% of survivors (p < 0.001). Heart failure, coronary atherosclerosis, and cardiac dysrhythmia were more common in non-survivors (35.1%, 30.3%, and 45.1%, respectively) than in survivors (p < 0.001 for all). Acute kidney injury (AKI) was nearly three times as prevalent in non-survivors (49.7%) compared to survivors (19.7%) (p < 0.001). Respiratory failure was significantly associated with mortality, occurring in 71.2% of non-survivors versus 11.7% of survivors (p < 0.001). This strong association suggests that either respiratory complications arise more frequently in critically ill stroke patients or that preexisting conditions contribute to worse outcomes (Table 2).

**Table 2:** Comorbidities and procedure-related factors during index admission for acute stroke (non-survivors vs. survivors) MI: myocardial infarction; AKI: acute kidney injury; COPD: chronic obstructive pulmonary disease; CKD: chronic kidney disease; UTI: urinary tract infection; PCI: percutaneous coronary intervention; ALL: acute lymphoblastic leukemia; CABG: coronary artery bypass graft; CVA: cerebrovascular accident; PAD: peripheral arterial disease; GI: gastrointestinal

Comorbidities	No Mortality (n=626,093), n (%)	Mortality (n=53,587), n (%)	Overall (n=679,680), n (%)	Test Statistic (df)	P value
Cardiac arrest	4,274 (0.7%)	6,399 (11.9%)	10,673 (1.6%)	χ² = 850.3 (1)	.001
Heart failure	127,929 (20.4%)	18,795 (35.1%)	146,724 (21.6%)	χ² = 482.2 (1)	.001
Coronary atherosclerosis	174,258 (27.8%)	16,220 (30.3%)	190,478 (28.0%)	χ² = 150.0 (1)	.001
Shock	10,385 (1.7%)	6,813 (12.7%)	17,198 (2.5%)	χ² = 1200.5 (1)	.001
Cardiac dysrhythmias	182,631 (29.2%)	24,151 (45.1%)	206,782 (30.4%)	χ² = 800.2 (1)	.001
AKI	123,359 (19.7%)	26,610 (49.7%)	149,969 (22.1%)	χ² = 1100.4 (1)	.001
COPD	84,425 (13.5%)	9,743 (18.2%)	94,168 (13.9%)	χ² = 250.0 (1)	.001
CKD	136,637 (21.8%)	15,354 (28.7%)	151,991 (22.4%)	χ² = 350.0 (1)	.001
Respiratory failure	73,041 (11.7%)	38,156 (71.2%)	111,197 (16.4%)	χ² = 2500.3 (1)	.001
Fluid and electrolyte disorders	202,213 (32.3%)	34,732 (64.8%)	236,945 (34.9%)	χ² = 3000.7 (1)	.001
Cardiogenic shock	4,745 (0.8%)	3,054 (5.7%)	7,799 (1.1%)	χ² = 400.0 (1)	.001
Tobacco use	858 (0.1%)	25 (0.0%)	883 (0.1%)	χ² = 65.0 (1)	.001
Alcohol abuse	29,287 (4.7%)	2,180 (4.1%)	31,467 (4.6%)	χ² = 30.0 (1)	.001
Lipid disorders	384,668 (61.4%)	22,244 (41.5%)	406,912 (59.9%)	χ² = 1500.5 (1)	.001
Hypertension	372,177 (59.4%)	20,335 (37.9%)	392,512 (57.7%)	χ² = 2000.0 (1)	.001
Diabetes	252,532 (40.3%)	20,475 (38.2%)	273,007 (40.2%)	χ² = 7.2 (1)	0.007
Obesity	101,664 (16.2%)	6,377 (11.9%)	108,041 (15.9%)	χ² = 80.0 (1)	.001
Valvular heart disease	35,964 (5.7%)	3,000 (5.6%)	38,964 (5.7%)	χ² = 1.75 (1)	0.163
PAD	42,977 (6.9%)	3,467 (6.5%)	46,444 (6.8%)	χ² = 14.3 (1)	.001
GI Bleed	13,541 (2.2%)	4,170 (7.8%)	17,711 (2.6%)	χ² = 400.0 (1)	.001
Liver failure	5,112 (0.8%)	4,301 (8.0%)	9,413 (1.4%)	χ² = 350.0 (1)	.001
Thyroid disorders	100,455 (16.0%)	7,199 (13.4%)	107,654 (15.8%)	χ² = 60.0 (1)	.001
Anemia	23,817 (3.8%)	6,119 (11.4%)	29,936 (4.4%)	χ² = 500.0 (1)	.001
Coagulation disorders	48,635 (7.8%)	11,948 (22.3%)	60,583 (8.9%)	χ² = 700.0 (1)	.001
Cancer	58,504 (9.3%)	8,222 (15.3%)	66,726 (9.8%)	χ² = 300.0 (1)	.001
Depression	81,854 (13.1%)	4,506 (8.4%)	86,360 (12.7%)	χ² = 150.0 (1)	.001
Dementia and Neurocognitive disorders	83,974 (13.4%)	8,077 (15.1%)	92,051 (13.5%)	χ² = 40.0 (1)	.001
Complications of device	7,244 (1.2%)	1,859 (3.5%)	9,103 (1.3%)	χ² = 200.0 (1)	.001
Pneumonia	40,881 (6.5%)	16,356 (30.5%)	57,237 (8.4%)	χ² = 1000.0 (1)	.001
Vasopressors	7,309 (1.2%)	5,945 (11.1%)	13,254 (2.0%)	χ² = 600.0 (1)	.001
Pulmonary Circulatory disorders	24,456 (3.9%)	3,485 (6.5%)	27,941 (4.1%)	χ² = 100.0 (1)	.001
In hospital bleeding	1,032 (0.2%)	196 (0.4%)	1,228 (0.2%)	χ² = 30.0 (1)	<.001
In hospital vascular complications	505 (0.1%)	108 (0.2%)	613 (0.1%)	χ² = 15.0 (1)	.001
Conduction disorder	34,816 (5.6%)	3,320 (6.2%)	38,136 (5.6%)	χ² = 9.2 (1)	.001
PCI ALL	5,023 (0.8%)	1,012 (1.9%)	6,035 (0.9%)	χ² = 45.0 (1)	.001
CABG during admission	2,922 (0.5%)	544 (1.0%)	3,466 (0.5%)	χ² = 30.0 (1)	.001
AMI	33,141 (5.3%)	9,835 (18.4%)	42,976 (6.3%)	χ² = 1200.0 (1)	.001
COVID	14,898 (2.4%)	6,686 (12.5%)	21,584 (3.2%)	χ² = 900.0 (1)	.001

**Figure 1:**
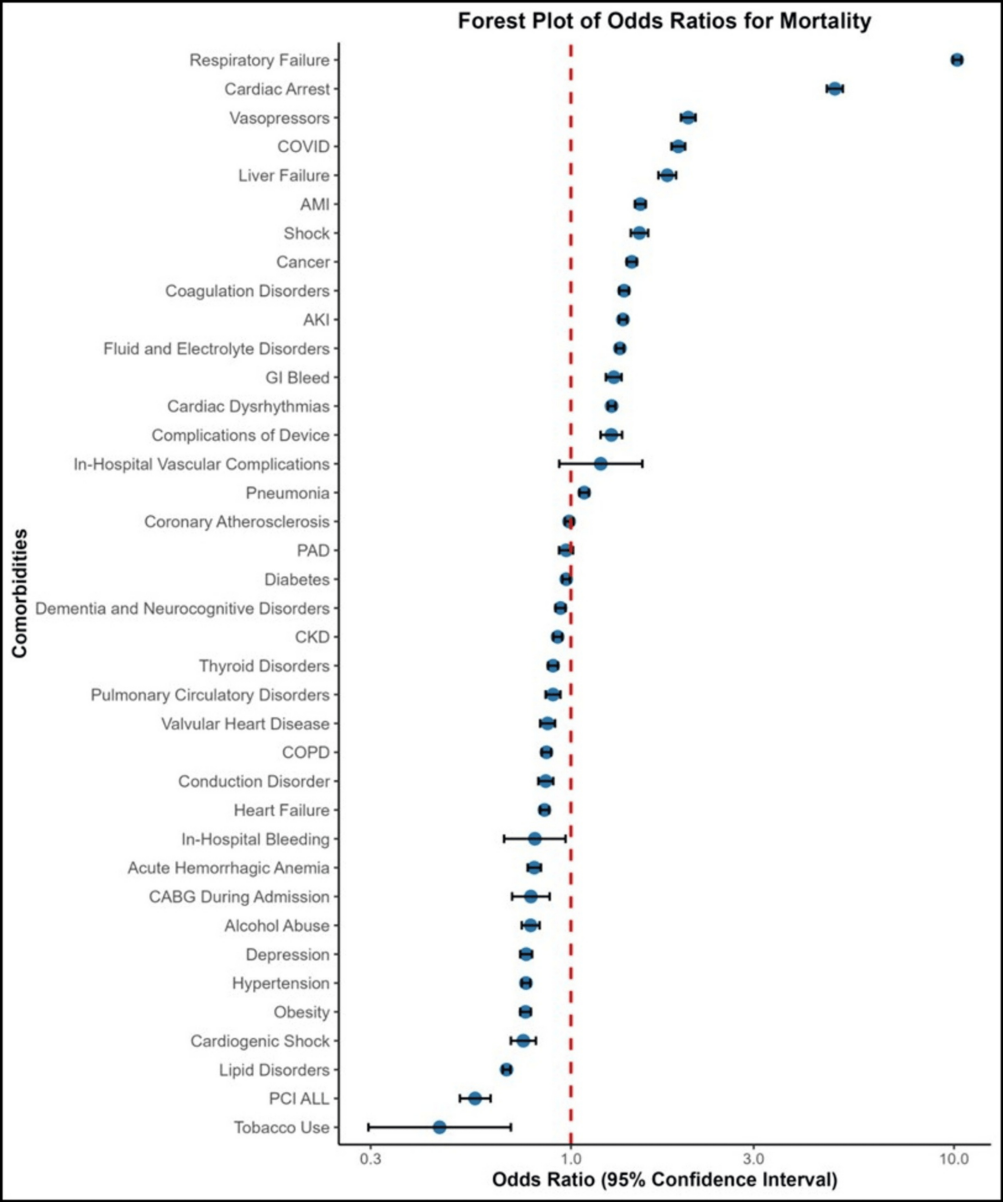
Forest plot of odds ratios for in-hospital mortality among acute stroke patients COVID: coronavirus disease; AKI: acute kidney injury; COPD: chronic obstructive pulmonary disease; PAD: peripheral artery disease; CKD: chronic kidney disease; PCI: percutaneous coronary intervention; ALL: acute lymphoblastic leukemia; CABG: coronary artery bypass graft; AMI: acute myocardial infarction

Metabolic imbalances, such as fluid and electrolyte disorders, were more frequent among non-survivors (64.8%) than survivors (32.3%) (p < 0.001). Tobacco use, although infrequent overall, was significantly more common in non-survivors (p < 0.001). Hypertension and lipid disorders were highly prevalent but significantly more common among non-survivors (p < 0.001). Diabetes, while prevalent in both groups, showed only a modest difference between non-survivors (40.3%) and survivors (38.2%) (p = 0.007). This suggests that while diabetes contributes to overall stroke risk, it may be a weaker predictor of in-hospital mortality compared to conditions such as heart failure or respiratory failure (Table 3 and Figure 1).

**Table 3:** Multivariable logistic regression analysis for predicting mortality AKI: acute kidney injury; COPD: chronic obstructive pulmonary disease; PAD: peripheral artery disease; CKD: chronic kidney disease; PCI: percutaneous coronary intervention; ALL: acute lymphoblastic leukemia; CABG: coronary artery bypass graft; AMI: acute myocardial infarction

Variable	OR (95% CI)	Test Statistic (Wald z)	P value
Age (years)			
≥75 (reference)	1 (reference)	–	–
18–44	0.334 (0.314–0.355)	z = -15.0	.001
45–64	0.481 (0.463–0.498)	z = -22.0	.001
65–74	0.643 (0.625–0.660)	z = -18.0	.001
Sex			
Male (reference)	1 (reference)	–	–
Female	0.973 (0.952–0.995)	z = -2.2	.014
Insurance Status			
Medicaid (reference)	1 (reference)	–	–
Medicare	0.990 (0.947–1.035)	z = -0.5	.652
Private insurance	0.970 (0.928–1.015)	z = -1.3	.185
Self‑pay	1.346 (1.255–1.443)	z = 8.5	.001
No charge	0.810 (0.623–1.054)	z = -1.5	0.117
Other	1.281 (1.196–1.373)	z = 6.8	.001
Hospital Bed Size			
Small (reference)	1 (reference)	–	–
Medium	1.090 (1.052–1.129)	z = 4.5	.001
Large	1.165 (1.129–1.203)	z = 6.0	.001
Control/Ownership of Hospital			
Government (reference)	1 (reference)	–	–
Private non‑profit	1.008 (0.976–1.042)	z = 0.25	.618
Private for‑profit	0.867 (0.829–0.906)	z = -7.0	.001
Hospital Designation			
Large metropolitan, ≥1 million residents	1.150 (1.124–1.177)	z = 9.0	.001
Small metropolitan, ≤1 million residents	1.189 (1.131–1.249)	z = 8.5	.001
Micropolitan	1.787 (1.652–1.934)	z = 16.5	.001
Comorbidities			
Cardiac arrest	4.886 (4.656–5.126)	z = 40.0	.001
Heart failure	0.853 (0.831–0.877)	z = -12.0	.001
Coronary atherosclerosis	0.989 (0.965–1.014)	z = -0.8	.380
Shock	1.509 (1.433–1.590)	z = 15.0	.001
Cardiac dysrhythmias	1.277 (1.249–1.307)	z = 16.0	.001
AKI	1.367 (1.335–1.401)	z = 18.0	.001
COPD	0.863 (0.839–0.888)	z = -14.0	.001
CKD	0.923 (0.899–0.948)	z = -9.0	.001
Respiratory failure	10.192 (9.938–10.451)	z = 60.0	.001
Fluid and electrolyte disorders	1.342 (1.310–1.374)	z = 20.0	.001
Cardiogenic shock	0.751 (0.697–0.810)	z = -7.0	.001
Tobacco use	0.454 (0.296–0.697)	z = -3.0	.001
Alcohol abuse	0.785 (0.744–0.828)	z = -8.0	.001
Lipid disorders	0.679 (0.664–0.695)	z = -25.0	.001
Hypertension	0.763 (0.744–0.783)	z = -30.0	.001
Diabetes	0.972 (0.950–0.995)	z = -2.1	.016
Obesity	0.761 (0.737–0.786)	z = -18.0	.001
Valvular heart disease	0.869 (0.831–0.909)	z = -8.0	.001
PAD	0.972 (0.931–1.013)	z = -1.3	.180
GI Bleed	1.294 (1.234–1.356)	z = 10.0	.001
Liver failure	1.784 (1.692–1.882)	z = 20.0	.001
Thyroid disorders	0.898 (0.871–0.925)	z = -10.0	.001
Acute hemorrhagic anemia	0.802 (0.771–0.835)	z = -11.0	.001
Coagulation disorders	1.376 (1.337–1.417)	z = 19.0	.001
Cancer	1.441 (1.398–1.486)	z = 15.0	.001
Depression	0.764 (0.737–0.792)	z = -12.0	.001
Dementia and Neurocognitive disorders	0.939 (0.911–0.968)	z = -6.0	.001
Complications of device	1.275 (1.195–1.360)	z = 9.0	.001
Pneumonia	1.083 (1.052–1.114)	z = 5.0	.001
Vasopressors	2.024 (1.937–2.115)	z = 17.0	.001
Pulmonary Circulatory disorders	0.898 (0.859–0.939)	z = -8.0	.001
In hospital bleeding	0.805 (0.669–0.968)	z = -2.3	.021
In hospital vascular complications	1.196 (0.932–1.536)	z = 1.1	.160
Conduction disorder	0.859 (0.822–0.899)	z = -9.0	.001
PCI ALL	0.562 (0.513–0.617)	z = -12.5	.001
CABG during admission	0.786 (0.702–0.880)	z = -7.5	.001
COVID	1.907 (1.830–1.987)	z = 13.0	.001
AMI	1.518 (1.469–1.568)	z = 14.0	.001

## Discussion

These findings emphasize the importance of early risk stratification, particularly for older patients and those with pre-existing comorbidities, and those who develop acute complications during hospitalization. This distinction is crucial for identifying independent predictors of mortality and guiding targeted interventions, such as optimized cardiac and respiratory support. Ensuring equitable healthcare access remains critical in improving survival outcomes for high-risk stroke patients (cerebral infarction). The significantly higher median age among non-survivors (73 years vs. 71 years, p < 0.001) aligns with prior research showing that advanced age is a critical risk factor for stroke-related mortality [10]. Additionally, the slightly higher proportion of women among non-survivors (48.8% vs. 47.2%, p < 0.001) suggests that sex-related physiological and hormonal differences may influence stroke outcomes, a trend previously observed in epidemiological studies [11]. The lower proportion of elective admissions among non-survivors (2.9% vs. 3.9%, p < 0.001) may reflect the emergent nature of stroke-related hospitalizations, which are often associated with poorer prognoses when not managed immediately [12].

Insurance status emerged as a notable factor in stroke outcomes. Medicare was the predominant insurance type among both groups, but its higher representation among survivors (69.8% vs. 64.8%, p < 0.001) could suggest better access to follow-up care and rehabilitation. Conversely, private insurance coverage was lower among non-survivors (13.7% vs. 18.2%, p < 0.001), which may indicate disparities in healthcare access and quality, consistent with previous literature [13]. The significantly higher median hospitalization cost among non-survivors ($122,763.67 vs. $56,497, P < 0.001) and longer length of stay (seven days vs. four days, p < 0.001) reinforce the economic burden associated with severe stroke cases requiring intensive interventions [14].

Hospital characteristics also played a crucial role in patient outcomes. The "Extreme" likelihood of mortality classification for 84.6% of non-survivors compared to 20.1% of survivors (p < 0.001) underscores the prognostic value of early risk stratification in stroke patients. Additionally, large hospitals had a slightly higher proportion of non-survivors (62.9% vs. 57.5%, p < 0.001), which may reflect the referral of more severe cases to tertiary care centers. These hospitals often serve as specialized centers handling complex patients with multiple comorbidities, advanced age, or severe stroke presentations requiring intensive interventions. Moreover, they are more likely to manage transfers from smaller facilities, where patients may have already experienced clinical deterioration before admission. While our analysis adjusted for case mix using demographic factors and comorbidities, unmeasured variables such as stroke severity at presentation and pre-hospital delays may still contribute to the observed mortality differences. While private non-profit hospitals accounted for most admissions (76.7% overall), the lack of significant variation in hospital ownership patterns suggests that institutional factors may not independently influence mortality rates [15].

The prevalence of comorbidities significantly impacted survival outcomes. Cardiac conditions, including heart failure (35.1% vs. 0.7%, p < 0.001), coronary atherosclerosis (30.3% vs. 0.7%, p < 0.001), and cardiac dysrhythmia (45.1% vs. 0.7%, p < 0.001), were markedly higher among non-survivors, aligning with existing research linking cardiovascular disease to stroke mortality [16]. AKI (49.7% vs. 19.7%, p < 0.001) and respiratory failure (71.2% vs. 11.7%, p < 0.001) were also strongly associated with increased mortality, highlighting the systemic complications that exacerbate stroke severity. Moreover, metabolic imbalances (64.8% vs. 32.3%, p < 0.001) and tobacco use (p < 0.001) further emphasize the role of modifiable risk factors in stroke prognosis [17].

Hypertension and lipid disorders, though prevalent in both groups, were significantly higher in non-survivors (p < 0.001), reinforcing their role as major contributors to cerebrovascular events [18]. Interestingly, diabetes, despite being common, showed only a modest difference between groups (40.3% vs. 38.2%, p = 0.007), suggesting that while diabetes increases stroke risk, other acute factors may play a more significant role in determining survival.

Collectively, these findings underscore the need for targeted management strategies focusing on cardiovascular health, early risk stratification, and equitable healthcare access to improve stroke outcomes. However, it is important to acknowledge the inherent limitations of claims data, including the lack of clinical stroke severity measures such as the NIHSS. Instead, severity was inferred using coding-based proxies like mortality risk and loss-of-function subclassifications, which may not fully capture the neurological impact of stroke. Additionally, coding errors and variations in documentation practices could introduce misclassification biases, potentially affecting the accuracy of comorbidity identification and outcome assessment. These factors should be considered when interpreting the relationship between stroke severity, comorbidities, and mortality risk. Future research should explore the impact of early intervention programs mitigating these disparities and improving survival rates in high-risk populations.

## Conclusions

This study provides critical insights into the factors influencing survival outcomes in acute stroke patients. The findings suggest that older age, comorbid conditions, and hospital-related factors significantly impact mortality rates. Higher healthcare costs and longer hospital stays among non-survivors emphasize the economic burden of severe stroke cases. Additionally, disparities in insurance coverage and comorbidity prevalence highlight the need for enhanced preventive strategies, early intervention, and improved healthcare accessibility. These findings can inform targeted interventions and policy decisions aimed at optimizing stroke care, particularly for high-risk populations. However, the reliance on administrative claims data, which lack detailed clinical measures such as the NIHSS and may include coding inaccuracies, presents a limitation that could affect the generalizability of the results. Future research should incorporate clinical parameters to validate these findings, refine risk stratification models, and further explore modifiable risk factors to improve prognosis and reduce mortality rates among stroke patients.
